# *BRCA1*, *BRCA2* and *PALB2* mRNA Expression as Prognostic Markers in Patients with Early Breast Cancer

**DOI:** 10.3390/biomedicines12061361

**Published:** 2024-06-19

**Authors:** Ina Shehaj, Slavomir Krajnak, Katrin Almstedt, Yaman Degirmenci, Sophia Herzog, Antje Lebrecht, Valerie Catherine Linz, Roxana Schwab, Kathrin Stewen, Walburgis Brenner, Annette Hasenburg, Marcus Schmidt, Anne-Sophie Heimes

**Affiliations:** Department of Obstetrics and Gynecology, University Medical Center, Johannes Gutenberg-University Mainz, 55131 Mainz, Germanymarcus.schmidt@unimedizin-mainz.de (M.S.); anne-sophie.heimes@unimedizin-mainz.de (A.-S.H.)

**Keywords:** mRNA expression, *BRCA1*, *BRCA2*, *PALB2*, metagene, breast cancer

## Abstract

Breast cancer (BC) poses a challenge in establishing new treatment strategies and identifying new prognostic and predictive markers due to the extensive genetic heterogeneity of BC. Very few studies have investigated the impact of mRNA expression of these genes on the survival of BC patients. Methods: We examined the impact of the mRNA expression of breast cancer gene type 1 (*BRCA1*), breast cancer gene type 2 (*BRCA2*), and partner and localizer of *BRCA2* (*PALB2*) on the metastasis-free survival (MFS) of patients with early BC using microarray gene expression analysis. Results: The study was performed in a cohort of 461 patients with a median age of 62 years at initial diagnosis. The median follow-up time was 147 months. We could show that the lower expression of *BRCA1* and *BRCA2* is significantly associated with longer MFS (*p* < 0.050). On the contrary, the lower expression of PALB2 was correlated with a shorter MFS (*p* = 0.049). Subgroup survival analysis identified the prognostic influence of mRNA expression for *BRCA1* among patients with luminal-B-like BC and for *BRCA2* and *PALB2* in the subset of patients with luminal-A-like BC (*p* < 0.050). Conclusions: According to our observations, *BRCA1*, *BRCA2*, and *PALB2* expression might become valuable biomarkers of disease progression.

## 1. Introduction

Breast cancer (BC) is, with increasing incidence, the most common cancer among women worldwide [1]. Its extensive genetic heterogeneity poses a challenge to establishing new treatment strategies [2]. To overcome the obstacles of the prediction of prognosis in terms of heterogeneity, it is crucial to understand how molecular and genetic characteristics affect recurrence and therapy resistance [3]. 

Multiple studies have explored the specific roles of inherited high-penetrance pathogenic variants of genes such as breast cancer gene type 1 (*BRCA1*) and breast cancer gene type 2 (*BRCA2*) and of moderate-penetrance BC risk variants, particularly partner and localizer of *BRCA2* (*PALB2*), checkpoint kinase 2 (*CHEK2*), and ataxia teleangiectasia mutated (*ATM*) in the pathogenesis of BC [4]. *BRCA* tumor-suppressor genes have been shown to be associated with an increased risk of breast cancer [5]. The two BRCA proteins are physically and functionally linked by PALB2, which is a third tumor-suppressor protein that directly binds BRCA1 and BRCA2 in the homologous recombination (HR) pathway [6]. It is noteworthy that the interaction between *BRCA* genes and *PALB2* is essential for cell cycle checkpoint control, genome stability, HR, and tumor suppression [5,6]. A better understanding of the role of *BRCA* genes in the pathogenesis of BC has revolutionized cancer treatment through the development of new targeted therapies. 

The practice-changing results of many studies identified not only the role of *BRCA 1*, *BRCA2* and *PALB2* gene mutations in the pathogenesis of breast and ovarian cancer but also served to implement new targeted therapies such as poly (ADP-ribose) polymerase inhibitors (PARPi). Based on the results of the OlympiAD and EMBRACA trials, two PARP inhibitors, Olaparib and Talazoparib, respectively, have been approved for the treatment of BRCAm carriers with metastatic HER2-negative BC [7,8]. Pursuing this further, OlympiA showed that BRCAm carriers with early-stage breast cancer can benefit from a PARP inhibitor [9]. However, one of the major challenges in the current therapy of BC is the lack of effective biomarkers for drug response and sensitivity. Preclinical and clinical studies have investigated the impact of BRCA1 levels on the sensitivity of DNA-damaging agents, such as cisplatin, and of antimicrotubule agents, such as paclitaxel [10,11,12].

Preclinical data have shown that *BRCA1* overexpression in human BC cell lines has resulted in increased resistance to DNA-damaging chemotherapy [13]. 

However, very few studies have investigated the role of messenger ribonucleic acid (mRNA) expression of *BRCA1/2* and *PALB2* on the survival of patients with BC and the relationship between BRCA1 and BRCA2 expression and response to chemotherapy [12,14,15,16,17]. A study by Green et al. demonstrated that a low level of *BRCA1* expression was associated with high numbers of CD8+ TILs, higher grades, high proliferation indexes, and poor survival in patients with BC (all *p* ≤ 0.010) [15]. Contrary to that, Margeli et al. showed that low levels of *BRCA1* mRNA and positive progesterone receptors in BC treated with anthracycline-based chemotherapy are associated with significantly longer survival (all *p* < 0.005) [12]. In another study, the association between the T cell activation status and death risk in patients with a low *BRCA1* expression level was statistically significant, proving the impact of *BRCA1* expression on the survival of patients with BC [14]. 

Published data suggest that there is a clinically significant relation between *BRCA1/2* mRNA expression and the prognosis of patients with BC. Therefore, the identification of new biomarkers could help establish survival prediction models and facilitate the development of other effective therapeutic strategies. 

Despite all advancements in the diagnosis and therapy of BC, the role of certain mutations and biomarkers remains unclear, and we still face many challenges in breast cancer research on a molecular basis. The aim of this study was to investigate the impact of mRNA expression of *BRCA1*, *BRCA2*, and *PALB2* on the metastasis-free survival of patients with early BC. 

## 2. Materials and Methods

### 2.1. Patient’s Characteristics

In the present study, 461 patients with early breast cancer treated at the Department of Gynecology and Obstetrics at the University Medical Center Mainz between 1986 and 2000 were included. Total RNA samples could be extracted from fresh frozen tumor tissue from each patient. All samples were assayed on Affymetrix Gene Chip in order to determine the mRNA expression values of *BRCA1*, *BRCA2*, and *PALB2*. Clinicopathological data such as age at initial diagnosis, histologic grade of differentiation, tumor size, nodal status, estrogen receptor (ER), progesterone receptor (PR), human epidermal growth factor receptor 2 (HER2), Ki-67, treatment details, and follow-up information were obtained from pathology reports and the breast cancer database of our department. 

A total of 200 patients with node-negative early BC received no further adjuvant therapy after surgery. Adjuvant therapy with tamoxifen was administered in 165 patients, while 96 patients received chemotherapy in the adjuvant setting.

### 2.2. Gene-Expression Analyses 

The following single genes were considered for gene expression analysis:

- *BRCA_1_204531_s_at.*
- *BRCA_1_211851_x_at.*
- *BRCA_2_214727_at.*
- *BRCA_2_208368_s_at.*
- *PALB2 219530_at.*

The metagenes of *BRCA1* and *BRCA2* were calculated for each sample as the average expression of all genes in the gene cluster. 

The mRNA expression analyses for *PALB2* and for metagenes *BRCA1* and *BRCA2* were performed with HG-U133A arrays (Affymetrix, Santa Clara, CA, USA), as previously described [18,19]. The median mRNA expression of the above-mentioned probe sets was used as the cut-off value. All patients included in this study were subdivided into two groups based on low and high levels of mRNA expression in *BRCA1*, *BRCA2* metagenes, and *PALB2*, using the median mRNA expression as the cut-off value. 

### 2.3. Statistical Analysis

Statistical analyses were performed using SPSS statistical software system version 27.0 (SPSS Inc., Chicago, IL, USA). A two-sided *p*-value < 0.05 was considered statistically significant. Kaplan–Meier survival analyses and univariable and multivariable Cox regression analyses were performed to examine the prognostic significance of *BRCA1*, *BRCA2*, and *PALB2* mRNA expression on metastasis-free survival (MFS). Subgroup analyses were performed in order to identify if there were any significant differences among *PALB2* and metagenene *BRCA1* and *BRCA2* levels of mRNA expression in different molecular subtypes. 

### 2.4. Subgroup Analyses

The molecular subtypes, also known as intrinsic subtypes, were assessed according to the three-gene model from Haibe-Kains et al., including the estrogen receptor gene (ESR1), HER2, and aurora kinase A (AURKA) [20].

The subgroup analyses were performed taking into consideration the following molecular subtypes:

1. Luminal-A-like: ESR1-positive, HER2-negative, and low proliferation (AURKA-low).
2. Luminal-B-like: ESR1-positive, HER2-negative, and high proliferation (AURKA-high).
3. HER2-positive.
4. Basal-like: ESR1 negative and HER2 negative.

## 3. Results

### 3.1. Patient and Tumor Characteristics 

We analyzed 461 patients with a median age of 62 years (range: 30–93) at initial diagnosis. The patient and tumor characteristics are summarized in Table 1. The median follow-up time was 147 months, ranging from 1 to 306 months. The majority of patients presented with ER-positive eBC 252 (73.5%) and PR-positive eBC 204 (59.5%). A total of 39 (8.5%) had an HER2-positive tumor. Among all our patients, 59 (11%) had TNBC. 

As shown in Table 1, the level of mRNA expression of *BRCA1* was significantly associated with tumor size, axillary nodal status, histological grade of differentiation, Ki-67 expression, and molecular subtypes. The mRNA expression of *BRCA2* was significantly associated with the histological grade of differentiation, Ki-67 expression, and molecular subtypes, whereas the mRNA expression of *PALB2* was associated with the axillary nodal status, age, ER, PR, and molecular subtypes (Table 1). 

### 3.2. Treatment

The surgical treatment of eBC was followed up in 165 patients from adjuvant therapy with tamoxifen and in 96 patients from adjuvant chemotherapy, either cyclophosphamide, methotrexate, fluorouracil, or epirubicin and cyclophosphamide. A total of 200 patients with node-negative early BC, received no further adjuvant therapy after surgery. Since the above-mentioned therapies were performed in an adjuvant setting and the Affymetrix microarray analyses were performed on fresh frozen tumor tissue (fresh frozen), they had no effect on the mRNA expression of *BRCA1* and *BRCA2* metagenes and also *PALB2*. 

### 3.3. Survival Analyses 

#### 3.3.1. Impact of *BRCA1* mRNA Expression on Survival

**Figure 1:**
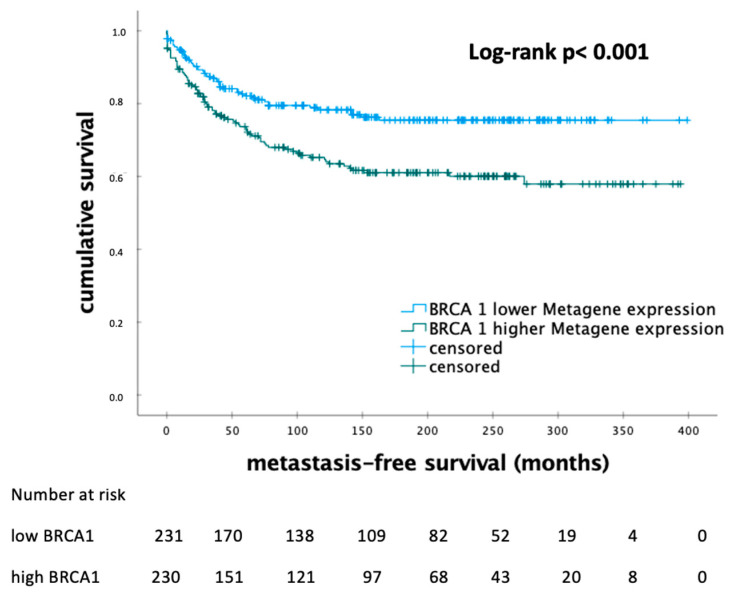
Kaplan–Meier analysis of metastasis-free survival in patients with early breast cancer according to the levels of *BRCA1* metagene mRNA expression. *BRCA1*—breast cancer 1; *p*-value < 0.05 is considered to be significant.

The number of patients with low or high expression levels of *BRCA1* was 231 and 230, respectively. The median MFS was 142.0 and 110.5 months in each subgroup, respectively. *BRCA1* mRNA expression levels correlated significantly with MFS (HR 1.803; 95% CI 1.269–2.561; *p* < 0.001). A lower BRCA1 mRNA expression was associated with a longer MFS in the Kaplan–Meier analysis (Figure 1). 

In the multivariable Cox regression analysis adjusted for age, tumor size, histological grade of differentiation, axillary nodal status, and the proliferation marker Ki-67, BRCA1 mRNA expression showed a significant influence on MFS (HR 1.611; 95% CI 1.047–2.479; *p* = 0.030) (Table 2). Similarly, tumor grading was also identified as an independent prognostic factor in the multivariable Cox regression analysis (HR 2.201; 95% CI 1.392–3.480; *p* < 0.001).

#### 3.3.2. Impact of *BRCA2* mRNA Expression on Survival

**Figure 2:**
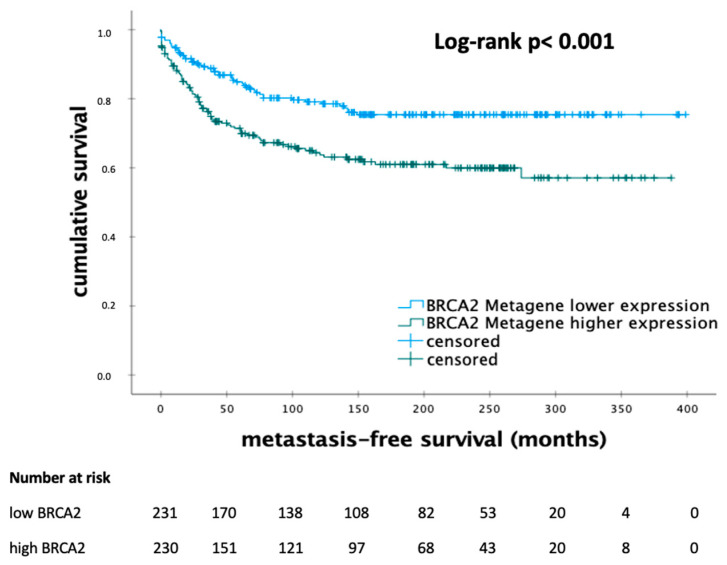
Kaplan–Meier analysis of metastasis-free survival in patients with early breast cancer according to the levels of *BRCA2* metagene mRNA expression. Abbreviations: *BRCA2*—breast cancer 2; *p*-value < 0.05 is considered to be significant.

The Kaplan–Meier analysis indicated that breast cancer patients with higher mRNA expression of BRCA2 had a shorter MFS (*p* < 0.001) (Figure 2). In this cohort, the number of patients with low or high expression levels of BRCA2 was 230 and 231, respectively. The median MFS was 151.5 months in the subgroup with lower levels of BRCA2 mRNA expression and 99.0 months in the other group of patients.

In the univariable analysis, the mRNA expression of *BRCA2* correlated significantly with MFS (HR 1.881; 95% CI 1.324–2.673; *p* < 0.001). In contrast, in the multivariable Cox regression analysis adjusted for age, tumor size, histological grade of differentiation, axillary nodal status, and the proliferation marker Ki-67, *BRCA2* mRNA expression was not significantly associated with survival (HR 1.315; 95% CI 0.870–1.988; *p* = 0.194) (Table 3).

#### 3.3.3. Impact of *PALB2* mRNA Expression on the Survival

**Figure 3:**
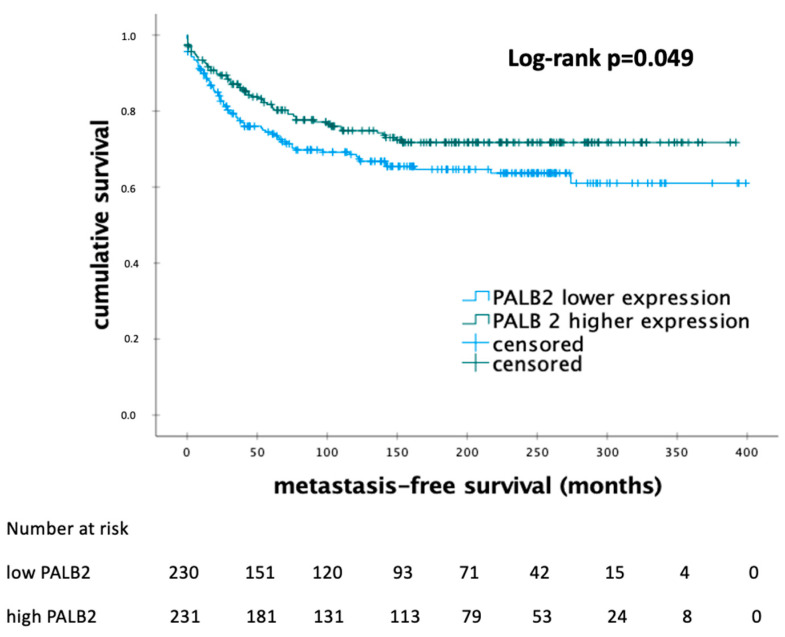
Kaplan–Meier analysis of metastasis-free survival in patients with early breast cancer according to the levels of *PALB2* mRNA expression. Abbreviations: *PALB2*—partner and localizer of *BRCA2*; *BRCA2*—breast cancer 2, *p*-value < 0.05 is considered to be significant.

The Kaplan–Meier analysis showed that 230 patients with lower levels of *PALB2* mRNA expression had a significantly shorter MFS compared to the patients with higher mRNA expression (115 vs. 148 months; *p* = 0.049) (Figure 3). 

The impact of *PALB2* mRNA expression on MFS could also be verified in the univariable Cox regression analysis (HR 0.710; 95% CI 0.504–1.001; *p* = 0.050). However, in the multivariable Cox regression analysis, *PALB2* mRNA expression failed to show a significant impact on MFS (HR 1.075; 95% CI 0.690–1.676; *p* = 0.748) (Table 4). As shown in Table 3, when adjusted for age, tumor size, histological grade of differentiation, axillary nodal status, and the proliferation marker Ki-67, only histological grading was found to be a prognostic factor related to MFS (HR 2.244; 95% CI 1.387–3.629; *p* < 0.001).

#### 3.3.4. Impact of *BCRA1* mRNA Expression on the Survival among Different Molecular Subtypes of Breast Cancer

The majority of patients (82.9%) with luminal-A-like BC showed a lower expression of *BRCA1* mRNA expression. Interestingly, no differences were observed in TNBC (lower vs. higher 25 vs. 26) (Table 5).

**Figure 4:**
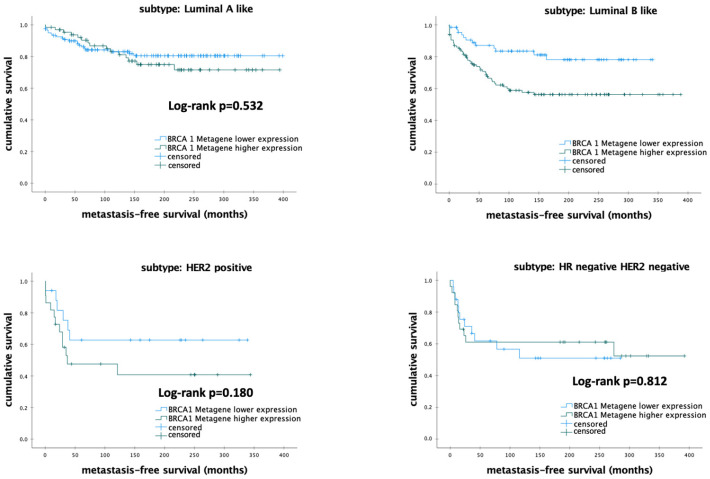
Kaplan–Meier analysis of metastasis-free survival in patients with early breast cancer according to *BRCA1* metagene mRNA expression. Abbreviations: *BRCA1*—breast cancer 1; luminal-A-like—estrogen receptor positive, HER2-negative, and low proliferation (AURKA-low); Luminal-B-like—estrogen receptor positive, HER2-negative, and high proliferation (AURKA-high); *p*-value < 0.05 is considered to be significant.

Further, subgroup analyses revealed that the MFS rates were significantly increased in patients with luminal-B-like BC (*p* = 0.003) (Figure 4). Nevertheless, there were no significant differences regarding MFS in other molecular subtypes (all *p* > 0.050).

The multivariable analysis showed a significant correlation between BRCA1 mRNA expression and MFS in the luminal-A-like subgroup of patients (HR 0.390; 95%-CI 0.229–0.666; *p* < 0.001) (Table 6).

#### 3.3.5. Impact of *BCRA2* mRNA Expression on the Survival among Different Molecular Subtypes of Breast Cancer

The correlation of *BRCA2* mRNA expression and MFS among different molecular subtypes is shown in Table 7.

**Figure 5:**
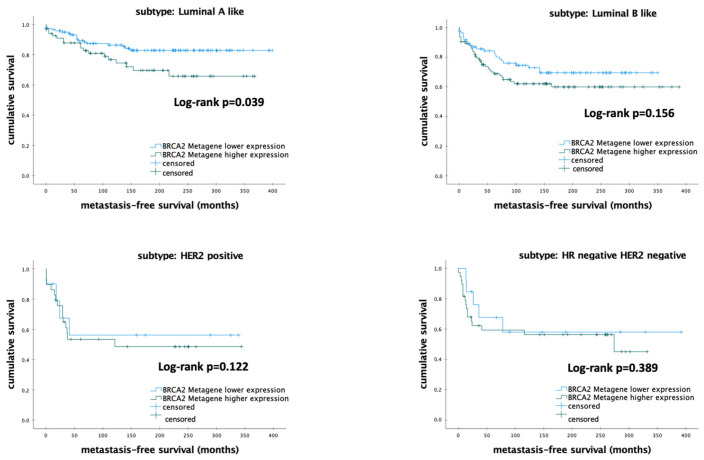
Kaplan–Meier analysis of metastasis-free survival in patients with early breast cancer according to *BRCA2* metagene mRNA expression. Abbreviations: *BRCA2*—breast cancer 2; luminal-A-like—estrogen receptor positive, HER2-negative, and low proliferation (AURKA-low); luminal-B-like—estrogen receptor positive, HER2-negative, and high proliferation (AURKA-high); *p*-value < 0.05 is considered to be significant.

Subgroup analyses indicated that in patients with luminal-A and HER-2-positive like BC, lower mRNA expression of *BRCA2* was associated with higher survival rate (*p* = 0.039) (Figure 5). There was no significant difference in other molecular subtypes (all *p* > 0.050).

Further subgroup analyses with univariable and multivariable Cox regression analyses revealed the results shown on Table 8. The level of mRNA expression of *BRCA2* correlated significantly with MFS among the patients with luminal A and Her2-positive BC in the univariate analyses. However, in the multivariable model, mRNA expression of *BRCA2* could be confirmed as an independent prognostic marker only in the patients with luminal-A-like BC (HR 0.375; 95%-CI 0.209–0.671; *p* < 0.001) (Table 8).

#### 3.3.6. Impact of *PALB2* mRNA Expression on the Survival among Different Molecular Subtypes of Breast Cancer

The correlation of *PALB2* mRNA expression and MFS among different molecular subtypes is shown on Table 9. 

**Figure 6:**
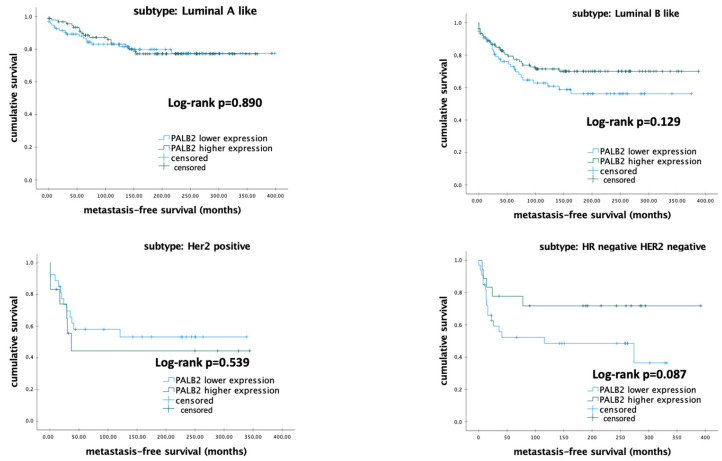
Kaplan–Meier analysis of metastasis-free survival in patients with early breast cancer according to *PALB2* Metagene mRNA expression.

Subgroup analyses showed no significant difference among different molecular subtypes regarding the impact of *PALB2* mRNA expression on MFS (all *p* > 0.050) (Figure 6).

Nevertheless, univariable and multivariable Cox regression analyses were conducted to evaluate the prognostic role of mRNA expression of *PALB2* among different molecular subtypes. The results of the Cox regression analyses are shown in Table 10. The level of mRNA expression of *PALB2* was identified as an independent prognostic marker related to MFS among the patients with luminal-A-like BC (HR 0.367; 95%-CI 0.216–0.625; *p* < 0.001) (Table 10). 

## 4. Discussion

The present study investigates the association between the level of mRNA expression of *BRCA1*, *BRCA2*, and *PALB2* and the MFS in eBC. To our knowledge, this is the first study designed to simultaneously investigate the impact of the mRNA expression of these three genes on the survival of patients with BC. 

Given the heterogeneous biology of BC, the different responses to therapy, and the high cost of treatments, there is an increasing interest in identifying the role of prognostic and treatment-predictive factors [21]. Inherited monoallelic mutations in *PALB2*, just like *BRCA1/2*, cause a high risk of breast cancer and also increase the risks of other cancers [4]. However, the role of messenger ribonucleic acid (mRNA) expression of different genes in the prognosis of BC is incompletely understood [22]. 

In our study, an increased level of mRNA expression of *BRCA1* and *BRCA2* was associated with several factors that are generally related to poor survival in breast cancer, such as tumor size, axillary nodal status, histological grade of differentiation, Ki-67 expression, and molecular subtypes. However, this observation could not be confirmed in the same analysis for *PALB2*.

Survival analysis showed a favorable MFS for low *BRCA1* mRNA expression in the whole cohort of included patients. Furthermore, a detailed analysis proved that the prognostic influence of *BRCA1* mRNA expression was most marked in the subset of patients with luminal-B-like BC. The cause of this finding may be the higher proliferation rate in this molecular subtype, which, together with genetic instability, may increase the need for more DNA damage repair. The observation of Gudas et al., suggesting that the upregulation of *BRCA1* expression by steroid hormones is caused indirectly by increasing proliferation of breast cancer cells, could also support our results [23].

Prior studies that have addressed the prognostic significance of *BRCA1* mRNA expression have shown conflicting results [12,14,15]. Our results are in line with the results of Margeli et al., who showed that low levels of *BRCA1* mRNA and a positive progesterone receptor in BC treated with anthracycline-based chemotherapy are associated with significantly longer survival [12]. An important difference in both studies is the fact that our patients did not receive any kind of therapy before surgery. These findings are confirmed by the results of a study by Lu et al., proving the impact of *BRCA1* expression on survival. The authors assessed the association between the T cell activation status and death risk according to the *BRCA1* expression level. An over 50% reduction in the risk of mortality was observed in patients with a low *BRCA1* expression level and with an intermediate T cell activation score and in patients with a high T cell activation score compared to patients with a low T cell activation score. Patients in the activation group lived over 8 years longer than those in the exhaustion group if they had a low level of *BRCA1* expression. In contrast, no significant association was observed between the T cell activation score and the risk of mortality in patients with a high level of *BRCA1* expression [14]. Contrary to this report, in another study, a low level of *BRCA1* expression has been found to be associated with poor survival in patients with BC [15]. These controversial studies imply a compelling need to understand the mechanism of BRCA1 mRNA expression in BC.

Furthermore, the prognostic significance of *BRCA2* mRNA expression was explored in our study. Our investigations revealed a significant relation between *BRCA2* mRNA expression and MFS, and the survival rates were markedly higher in patients with lower *BRCA2* expression. The survival analysis among different molecular subtypes indicated that in luminal-A-like BC, lower mRNA expression is associated with higher survival rates.

Even though *BRCA2* plays an important role in DNA repair, the prognostic role of the level of *BRCA2* mRNA expression has not been proven in any studies till now. In a cohort of 671 patients without BRCA1/2 germline mutation, it was observed that *BRCA2* mRNA expression may not predict responses to anthracycline-based or taxane neoadjuvant chemotherapy, and its level was not associated with survival rates [17]. In an experimental study by Wu et al., *BRCA2* mRNA levels were significantly down-regulated in breast and ovarian cancer cell lines after exposure to various DNA-damaging agents [24]. The authors acknowledged that DNA-damaging agents reduce the rate of *BRCA2* mRNA and protein turnover in a p53-dependent manner.

*PALB2* is regarded as a BC risk gene and plays a crucial role in the DNA repair process, but the prognostic role of *PALB2* expression in BC remains unclear. Few reports have studied the relationship between *PALB2* and the survival of patients with cancer, especially BC [25,26,27]. In our study, the survival analysis showed that a low mRNA expression of *PALB2* correlated with shorter MFS. Similarly, Liu et al. assessed the impact of *PALB2* mRNA expression in 563 patients with primary BC and revealed that a low expression of *PALB2* mRNA is significantly associated with poor survival (HR = 1.79; 95% CI = 1.21–2.65; *p* = 0.003 [28]. 

Interestingly, the prognostic impact of mRNA expression of *PALB2* was identified in the subgroup of patients with luminal-A-like BC. Patients with lower levels of *PALB2* mRNA expression had a significantly shorter MFS compared to patients with higher mRNA expression. Our observations are consistent with reports in the literature and lend further support to the notion that *PALB2* mRNA expression is associated with the survival of patients with BC and has potential clinical applications as a predictive marker of prognosis.

The limitation of the present study is the retrospective design. Another potential weakness of our study is that our analyses were limited to gene expression data, and we do not have information regarding the *BRCA1/2* and *PALB2* gene germline mutations in the patients included in this study, as this is a historic cohort. 

The strength of this study is that we report the prognostic significance of mRNA expression of *BRCA1*, *BRCA2*, and *PALB2* in a large cohort; the inclusion of patients with a sufficient amount of fresh frozen tissue available for successful mRNA microarray analysis of gene expression; and the long-term follow-up.

In general, the current results support the independent prognostic role of mRNA expression of *BRCA1*, *BRCA2*, and *PALB2*. 

## 5. Conclusions

In general, we provided clinical evidence from a large cohort of 461 patients for the role of *BRCA1*, *BRCA2*, and *PALB2* mRNA levels as markers of MFS in patients with early BC. Our data support the independent prognostic role of mRNA expression of BRCA1, BRCA2, and PALB2. Our investigations revealed that the survival rates were markedly higher in patients with lower *BRCA1* or *BRCA2* expression and in patients with higher *PALB2* mRNA expression.

Moreover, the current study underscores the potential clinical application of mRNA expression of *BRCA1*, *BRCA2*, and *PALB2* in different survival predictor models as a marker of disease progression but also as a therapeutic indicator. As shown in other preclinical and clinical studies, the identification of such new biomarkers could also help to identify the role of mRNA expression on the drug response and drug resistance in order to facilitate the development of other effective therapeutic strategies. However, these results warrant further examination in prospective clinical trials, even in patients with known gene germline mutations.

## Figures and Tables

**Table 1:** Associations between median mRNA expression of *BRCA1/2* metagenes and *PALB2* and patient and tumor characteristics.

Characteristic	Patients Number (Percentage)	*BRCA1* Lower Expression	Higher Expression	*p*-Value	*BRCA2* Lower Expression	Higher Expression	*p*-Value	PALB2 Lower Expression	Higher Expression	*p*-Value
Tumor size										
pTis	5 (1.1)	0 (0)	5 (100)		1 (20.0)	4 (80.0)		2 (40.0)	3 (60.0)	
pT1	183 (39.8)	98 (53.6)	85 (46.4)		98 (53.6)	85 (46.4)		83 (45.4)	100 (54.6)	
pT2	214 (46.5)	110 (51.4)	104 (48.6)		107 (50.0)	107 (50.0)		105 (49.1)	109 (50.9)	
pT3	19 (4.1)	6 (31.6)	13 (68.4)		7 (36.8)	12 (63.2)		13 (68.4)	6 (31.6)	
pT4	39 (8.5)	16 (41.0)	23 (59.0)	0.042	17 (43.6)	22 (4.8)	0.322	26 (66.7)	13 (33.3)	0.067
Missing data	1 (0.2)									
Axillary nodal status										
pN0	253 (57.5)	119 (47.0)	134 (53.0)		134 (53.0)	119 (47.0)		95 (37.5)	158 (62.5)	
pN1	138 (31.4)	80 (58.0)	58 (42.0)		65 (47.1)	73 (52.9)		91 (65.9)	47 (34.1)	
pN2	49 (11.1)	20 (40.8)	5 (9.2)	0.049	22 (44.9)	27 (55.1)	0.396	30 (61.2)	19 (38.8)	<0.001
Missing data	21 (4.6)									
Histological grade										
I	62 (14.5)	40 (64.5)	22 (35.5)		37 (59.7)	25 (40.3)		30 (48.4)	32 (51.6)	
II	261 (60.8)	123 (47.1)	138 (52.9)		137 (52.5)	124 (47.5)		119 (45.6)	142 (54.4)	
III	106 (24.7)	47 (44.3)	59 (55.7)	0.027	40 (37.7)	66 (62.3)	0.009	61 (57.5)	45 (42.5)	0.115
Missing data	32 (6.9)									
Age at study entry										
<50 years	95 (20.6)	41 (43.2)	54 (56.8)		41 (43.2)	54 (56.8)		59 (62.1)	36 (37.9)	
≥50 years	366 (79.4)	190 (51.9)	176 (48.1)	0.128	189 (51.6)	177 (48.4)	0.141	171 (46.7)	195 (53.3)	0.008
Estrogen receptor status										
Positive	252 (73.5)	117 (46.4)	135 (53.6)		130 (51.6)	122 (48.4)		120 (47.6)	132 (52.4)	
Negative	91 (26.5)	46 (50.5)	45 (49.5)	0.500	39 (42.9)	52 (57.1)	0.153	55 (60.4)	36 (39.6)	0.036
Progesterone receptor status										
Positive	204 (59.5)	94 (46.1)	110 (53.9)		97 (47.5)	107 (52.5)		95 (46.6)	109 (53.4)	
Negative	139 (40.5)	69 (49.6)	70 (50.4)	0.517	72 (51.8)	67 (48.2)	0.440	80 (57.6)	59 (42.4)	0.046
Ki-67 (%) median										
≤20	199 (59.9)	114 (57.3)	85 (42.7)		119 (59.8)	80 (40.2)		86 (43.2)	113 (56.8)	
>20	133 (40.1)	48 (36.1)	85 (63.9)	<0.001	56 (42.1)	77 (57.9)	0.002	62 (46.6)	71 (53.4)	0.541
Missing data	129 (28.0)									
Molecular subtype										
Luminal A-like	189 (41.0)	123 (65.1)	66 (34.9)		120 (63.5)	69 (36.5)		95 (50.3)	94 (49.7)	
Luminal B-like	182 (39.5)	66 (36.3)	116 (63.7)		87 (47.8)	95 (52.2)		75 (41.2)	107 (58.8)	
Her2-positive	39 (8.5)	17 (43.6)	22 (56.4)		10 (25.6)	29 (74.4)		27 (69.2)	12 (30.8)	
Triple-negative	51 (11.1)	25 (49.0)	26 (51.0)	<0.001	13 (25.5)	38 (74.5)	<0.001	33 (64.7)	18 (35.3)	0.001

Abbreviations: *BRCA1*—breast cancer 1; mRNA—messenger ribonucleic acid; *PALB2*—partner and localizer of *BRCA2*; *BRCA2*—breast cancer 2; luminal-A-like—estrogen receptor positive, HER2-negative, and low proliferation (AURKA-low); luminal-B-like—estrogen receptor positive, HER2-negative, and high proliferation (AURKA-high); *p*-value < 0.05 is considered to be significant.

**Table 2:** Multivariable Cox regression analysis of *BRCA1* metagene mRNA expression for metastasis-free survival adjusted for age, tumor size, lymph node status, grade of differentiation, and the proliferation marker Ki-67.

Variables	Multivariable Analysis HR (95% CI)	*p*-Value
*BRCA1* mRNA expression	1.611 (1.047–2.479)	0.030
Age	1.167 (0.677–2.013)	0.578
Tumor size	1.479 (0.931–2.350)	0.097
Histological grade of differentiation	2.201 (1.392–3.480)	<0.001
Axillary nodal status	1.509 (0.977–2.330)	0.064
Ki-67	1.171 (0.739–1.857)	0.501

*BRCA1*—breast cancer 1; mRNA—messenger ribonucleic acid; CI—confidence interval; HR—hazard ratio; *p*-value < 0.05 is considered to be significant.

**Table 3:** Multivariable Cox regression analysis of *BRCA2* metagene mRNA expression for metastasis-free survival adjusted for age, tumor size, lymph node status, grade of differentiation, and the proliferation marker Ki-67.

Variables	Multivariable Analysis HR (95% CI)	*p*-Value
*BRCA2* mRNA expression	1.315 (0.870–1.988)	0.194
Age	1.180 (0.683–2.038)	0.552
Tumor size	1.512 (0.951–2.404)	0.080
Histological grade of differentiation	2.133 (1.347–3.376)	0.001
Axillary nodal status	1.429 (0.925–2.205)	0.107
Ki-67	1.271 (0.806–2.003)	0.302

Abbreviations: *BRCA2*—breast cancer 2; mRNA—messenger ribonucleic acid; CI—confidence interval; HR—hazard ratio; *p*-value < 0.05 is considered to be significant.

**Table 4:** Multivariable Cox regression analysis of *PALB2* mRNA expression for metastasis-free survival adjusted for age, tumor size, lymph node status, grade of differentiation, and the proliferation marker Ki-67.

Variables	Multivariable Analysis HR (95% CI)	*p*-Value
*PALB2* mRNA expression	1.075 (0.690–1.676)	0.748
Age	1.277 (0.703–2.141)	0.472
Tumor size	1.511 (0.939–2.433)	0.089
Histological grade of differentiation	2.244 (1.387–3.629)	<0.001
Axillary nodal status	1.245 (0.778–1.992)	0.360
Ki-67	1.507 (0.936–2.426)	0.092

Abbreviations: *PALB2*—partner and localizer of *BRCA2*; *BRCA2*—breast cancer 2; mRNA—messenger ribonucleic acid; CI—confidence interval; HR—hazard ratio; *p*-value < 0.05 is considered to be significant.

**Table 5:** Correlation of breast cancer subtypes and mRNA expression of *BRCA1* metagene.

Variables	mRNA Low Expression	mRNA High Expression
Luminal-A-like	123	66
Luminal-B-like	66	116
Her-2-positive	17	22
Triple-negative	25	26

Abbreviations: *BRCA1*—breast cancer 1; luminal-A-like—estrogen receptor positive, HER2-negative, and low proliferation (AURKA-low); luminal-B-like—estrogen receptor positive, HER2-negative, and high proliferation (AURKA-high).

**Table 6:** Association between *BRCA1* mRNA expression and metastasis-free survival using univariable and multivariable Cox regression analysis in different molecular subtypes.

Variables	Univariable Model HR (95%-CI)	*p*-Value	Multivariable Model HR (95%-CI)	*p*-Value
Luminal-A-like	0.500 (0.339–0.739)	<0.001	0.598 (0.391–0.914)	0.018
Luminal-B-like	1.098 (0.774–1.558)	0.599		
Her-2-positive	2.009 (1.221–3.305)	0.006	1.763 (1.036–2.999)	0.036
Triple-negative	1.750 (1.106–2.770)	0.017	1.531 (0.934–2.510)	0.934

Abbreviations: *BRCA1*—breast cancer 1; luminal-A-like—estrogen receptor positive, HER2-negative, and low proliferation (AURKA-low); luminal-B-like—estrogen receptor positive, HER2-negative, and high proliferation (AURKA-high); mRNA—messenger ribonucleic acid; CI—confidence interval; HR—hazard ratio; *p*-value < 0.05 is considered to be significant.

**Table 7:** Correlation of breast cancer subtypes and mRNA expression of *BRCA2* metagene.

Variables	mRNA Low Expression	mRNA High Expression
Luminal-A-like	120	69
Luminal-B-like	87	95
Her-2-positive	10	29
Triple-negative	13	38

Abbreviations: *BRCA2*—breast cancer 2; luminal-A-like—estrogen receptor positive, HER2-negative, and low proliferation (AURKA-low); luminal-B-like—estrogen receptor positive, HER2-negative, and high proliferation (AURKA-high).

**Table 8:** Association between *BRCA2* mRNA expression and metastasis-free survival using univariable and multivariable Cox regression analysis in different molecular subtypes.

Variables	Univariable Model HR (95%-CI)	*p*-Value	Multivariable Model HR (95%-CI)	*p*-Value
Luminal-A-like	0.512 (0.346–0.757)	<0.001	0.579 (0.380–0.883)	0.011
Luminal-B-like	1.170 (0.829–1.651)	0.371		
Her-2-positive	1.750 (1.056–2.899)	0.042	1.545 (0.904–2.640)	0.111
Triple-negative	1.564 (0.980–2.494)	0.050	1.377 (0.836–2.267)	0.209

Abbreviations: *BRCA2*—breast cancer 2; luminal-A-like—estrogen receptor positive, HER2-negative, and low proliferation (AURKA-low); luminal-B-like—estrogen receptor positive, HER2-negative, and high proliferation (AURKA-high); mRNA—messenger ribonucleic acid; CI—confidence interval; HR—hazard ratio; *p*-value < 0.05 is considered to be significant.

**Table 9:** Correlation of breast cancer subtypes and mRNA expression of *PALB2*.

Variables	mRNA Low Expression	mRNA High Expression
Luminal-A-like	120	69
Luminal-B-like	87	95
Her-2-positive	10	29
Triple-negative	13	38

Abbreviations: *PALB2*—partner and localizer of *BRCA2*; *BRCA2*—breast cancer 2; mRNA—messenger ribonucleic acid; *BRCA2*—breast cancer 2; luminal-A-like—estrogen receptor positive, HER2-negative, and low proliferation (AURKA-low); luminal-B-like—estrogen receptor positive, HER2-negative, and high proliferation (AURKA-high).

**Table 10:** Association between *PALB2* mRNA expression and metastasis-free survival using univariable and multivariable Coxregression analysis in different molecular subtypes.

Variables	Univariable Model HR (95%-CI)	*p*-Value	Multivariable Model HR (95%-CI)	*p*-Value
Luminal-A-like	0.452 (0.308–0.663)	<0.001	0.522 (0.343–0.793)	0.002
Luminal-B-like	1.278 (0.903–1.808)	0.166		
Her-2-positive	1.915 (1.155–3.176)	0.012	1.558 (0.906–2.680)	0.109
Triple-negative	1.754 (1.108–2.778)	0.017	1.421 (0.865–2.336)	0.165

Abbreviations: *PALB2*—partner and localizer of *BRCA2*; *BRCA2*—breast cancer 2; mRNA—messenger ribonucleic acid; luminal-A-like—estrogen receptor positive, HER2-negative, and low proliferation (AURKA-low); luminal-B-like—estrogen receptor positive, HER2-negative, and high proliferation (AURKA-high); CI—confidence interval; HR—hazard ratio; *p*-value < 0.05 is considered to be significant.

## Data Availability

Data are contained within the article.
